# Supplementary material for: Molecular Mechanism by Which Cobra Venom Cardiotoxins Interact with the Outer Mitochondrial Membrane
Source: Toxins (Basel). 2020 Jun 27;12(7):425. doi: 10.3390/toxins12070425 (PMC7404710; doi:10.3390/toxins12070425)
Supplement: Supplementary file 1 [file toxins-12-00425-s001.pdf]

Supplementary Materials: Molecular Mechanism by which Cobra Venom Cardiotoxins Interact with the Outer Mitochondrial Membrane

Feng Li , Indira H. Shrivastava, Paul Hanlon, Ruben K. Dagda and Edward S. Gasanoff

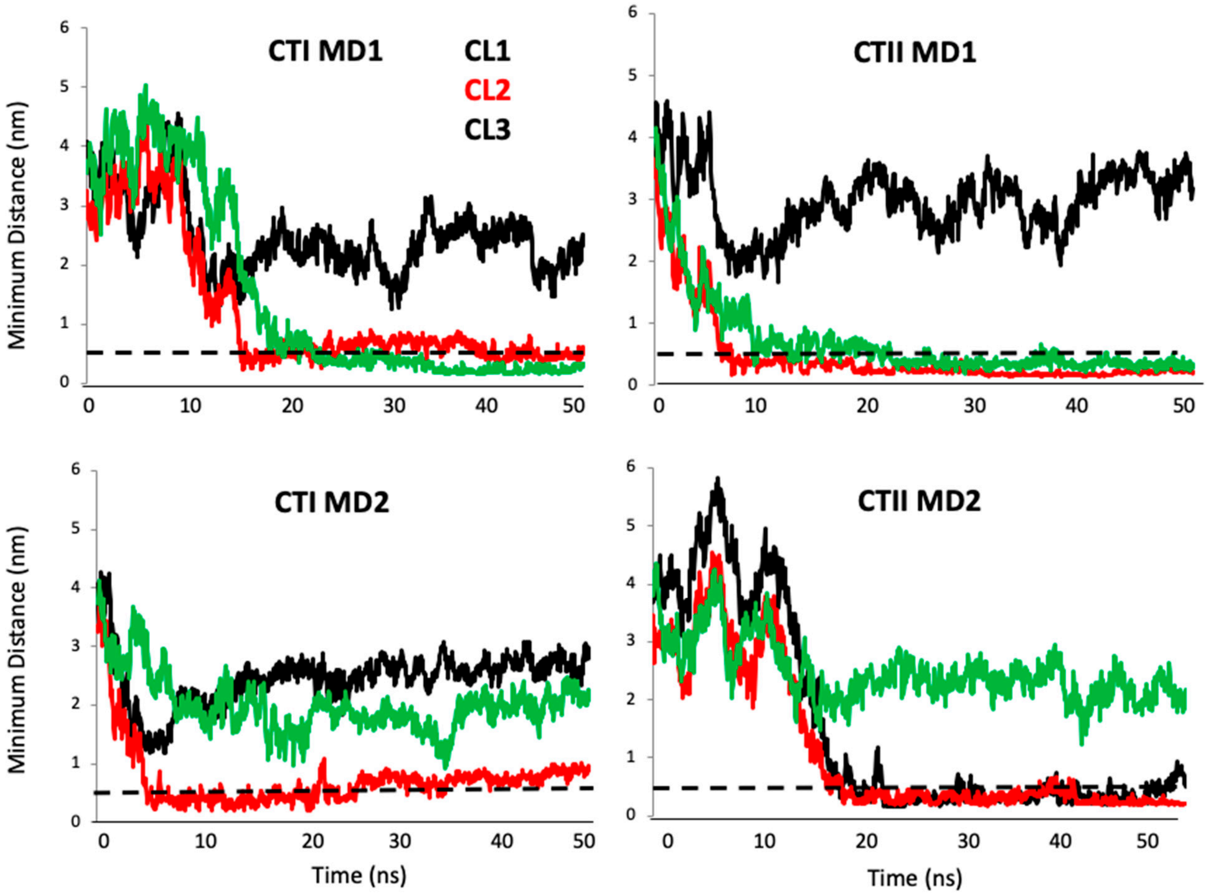

Figure S1. Time evolution analysis of the CTI (left panels top and bottom) and CTII (right panels top and bottom) interactions with the 3 CL molecules present in the bilayer. The dashed line indicates minimum distance (0.5nm) required for an intermolecular interaction. Note that in all of the simulations, once the toxin interacts with CL, it remains associated for the rest of the simulation time.

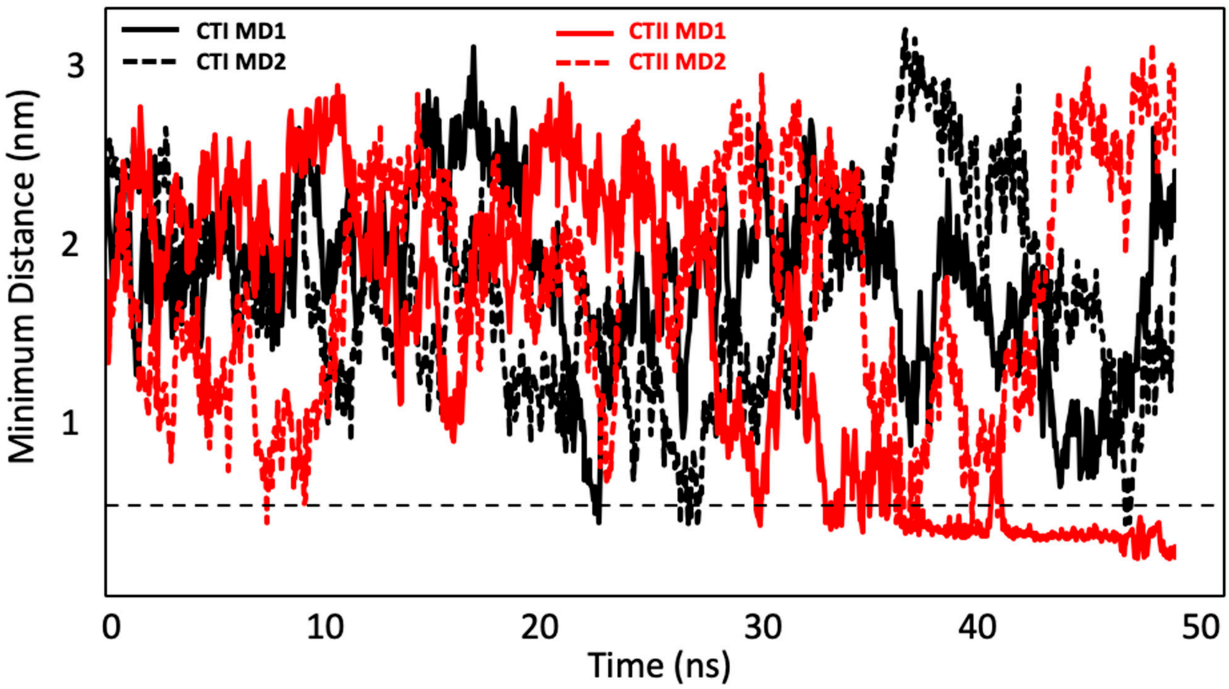

Figure S2. Time evolution analysis of the interaction of CTI and CTII with simulated membranes composed exclusively of POPC (no CL included) for two simulations for each cardiotoxin. Note that CTI is unable to significantly interact (come within 0.5nm) of the POPC membranes within 50ns (black curves), while CTII interacts only after 40ns in one simulation (red curve), while in the second simulation, it is unable to associate with the bilayer (red dashed curve).

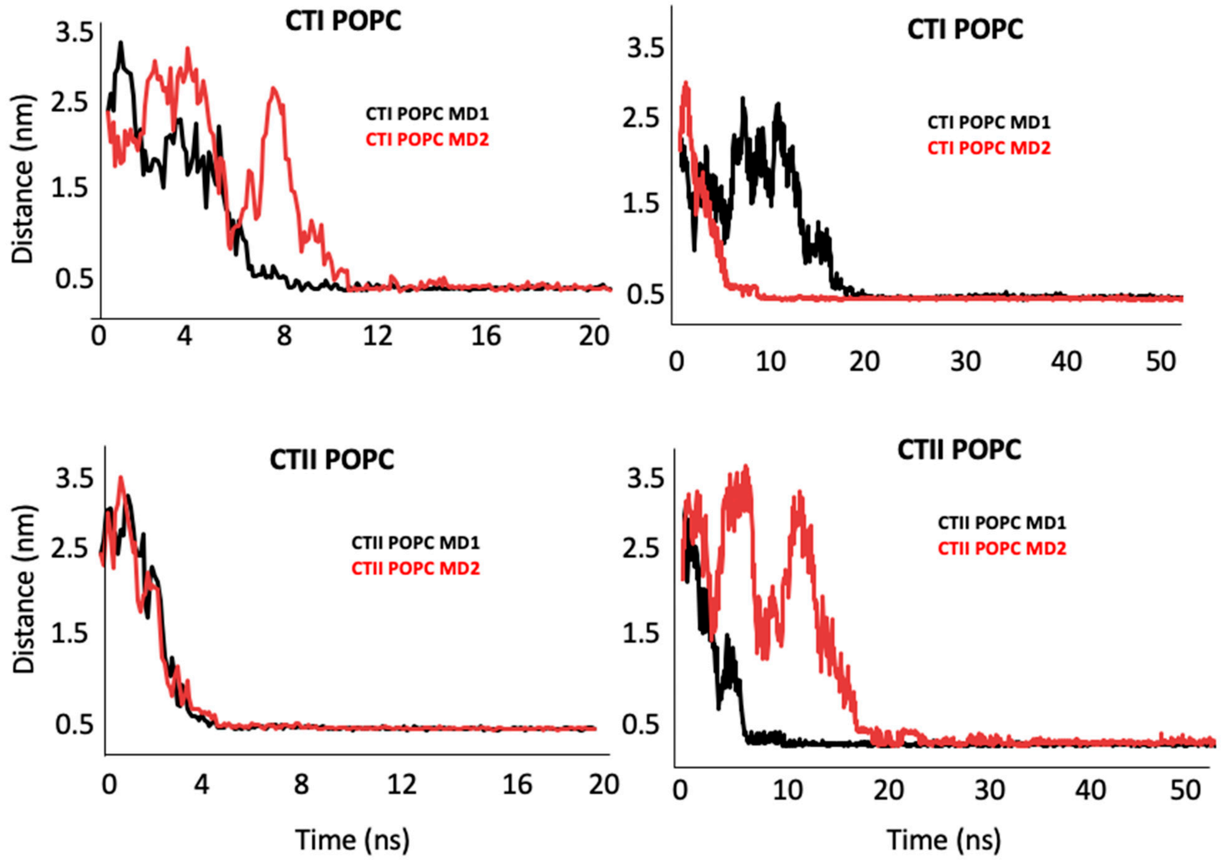

Figure S3. Time evolution of CTI/CTII interaction with POPC molecules in systems with CL. Minimum distance in 20ns runs (MD1, MD2) of any atom POPC in the bilayer and CTI (top left) or CTII (bottom left). Minimum distance in two 50ns runs (MD1, MD2) of any atom of POPC in the bilayer and CTI (top right) or CTII (bottom right).

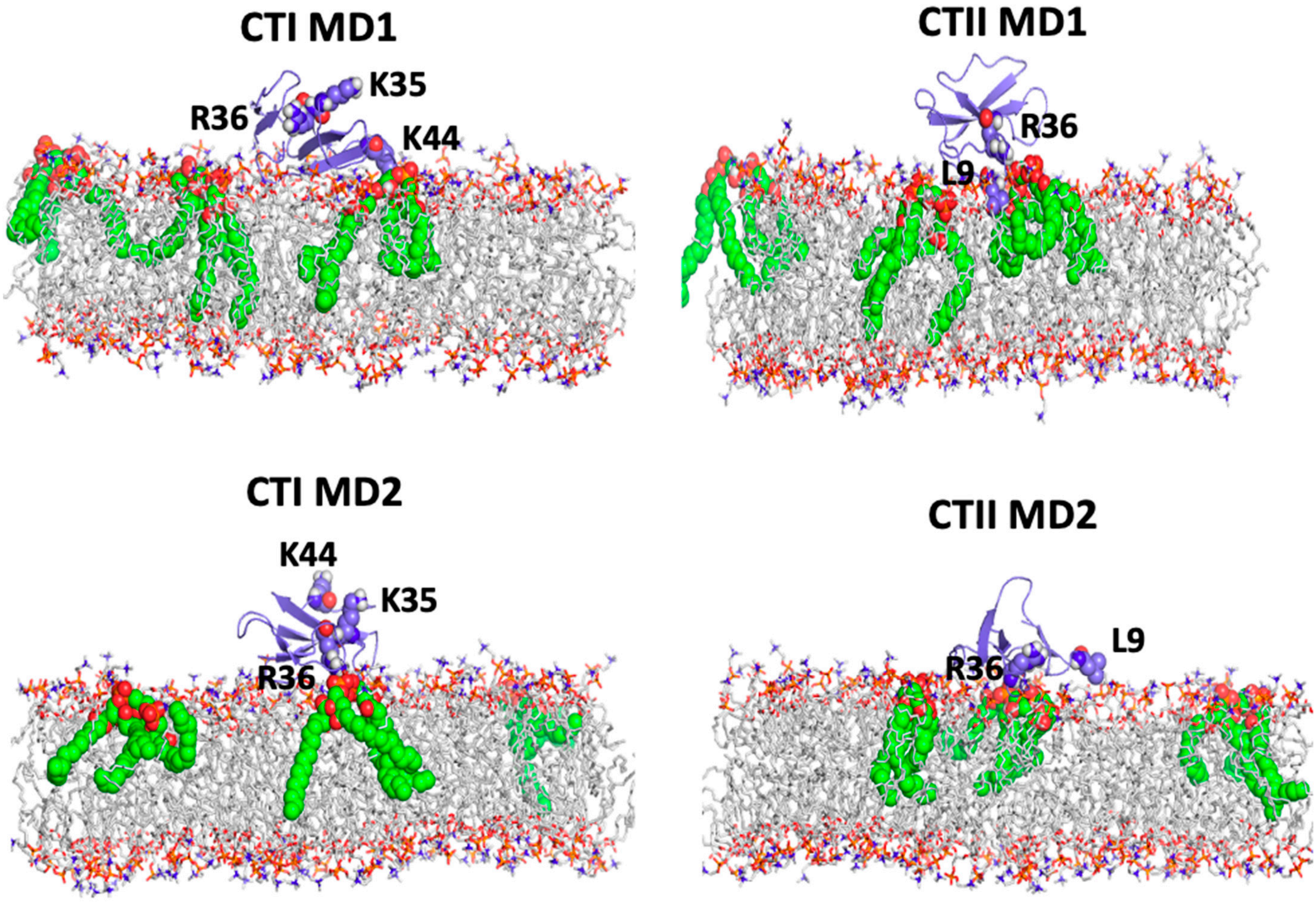

Figure S4. MD Snapshots of CTI/CTII systems with CL and POPC at 50 ns. (Left) CTI at membrane surface in MD1 (top), and MD2 (bottom). (Right) CTII at membrane surface in MD1 (top) and MD2 (bottom). In each panel, the protein is shown in ribbon diagram, the CL molecules in spheres, and the POPC molecules as stick representations. Few key residues are labelled for orientation.

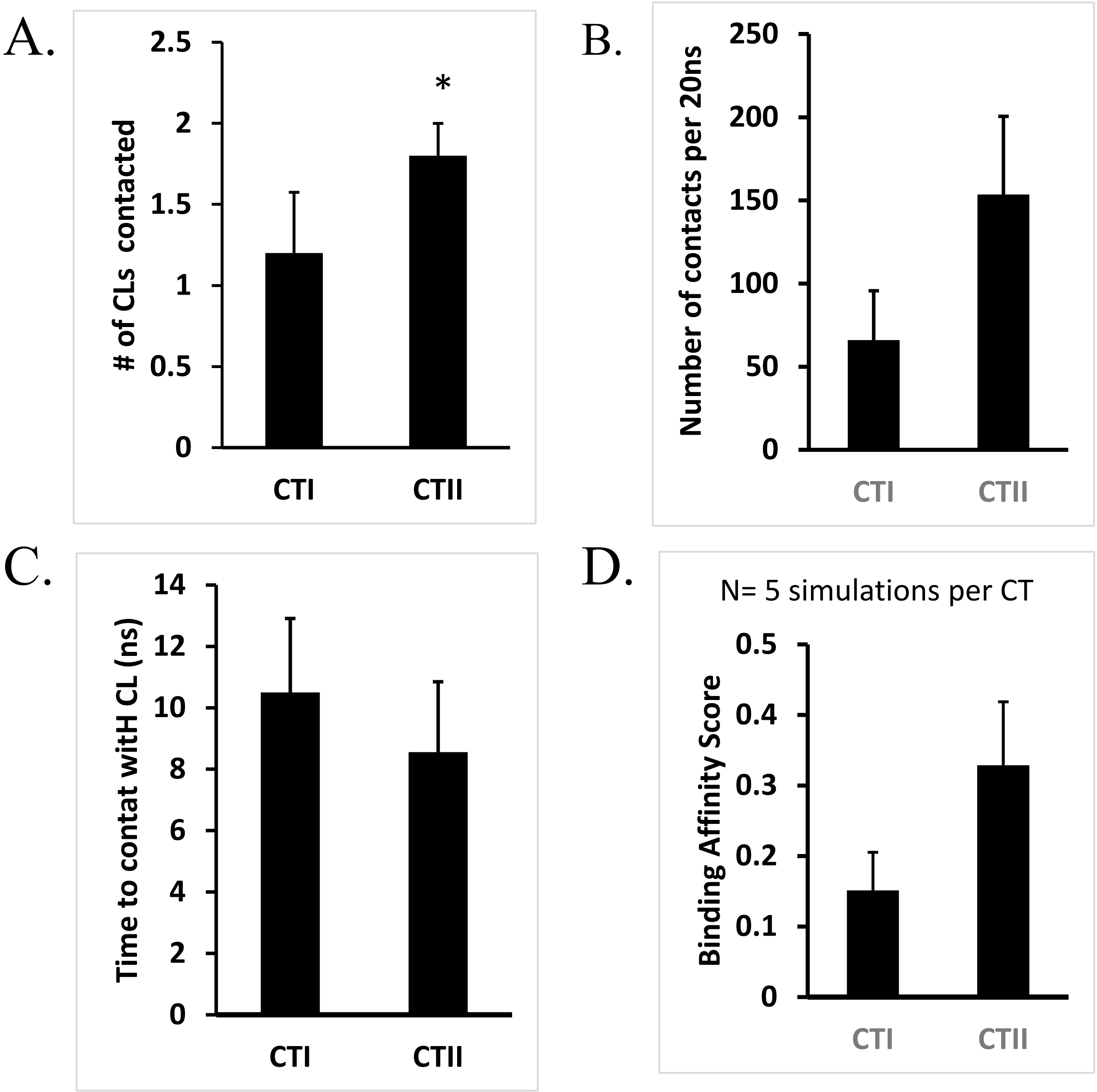

Figure S5. CTII shows trends in binding to simulated OMM more avidly than CTI. The ability for each cardiotoxin (CTI or CTII) to bind to POPC bilayers containing CLs was analyzed in 20ns and 50ns molecular dynamic (MD) runs by using four different metrics including the average number of CLs that bind to each cardiotoxin (**A**), the average number of contacts that each cardiotoxin makes with CLs within 20ns (**B**), the time that each cardiotoxin binds to CL in the simulated phospholipid bilayer (**C**) and the average binding affinity score for each cardiotoxin (**D**). Each bar graph shows the compiled average -/+ SEMs for each aforementioned metric (# of CLs contacted, average number of contacts, time to contact and affinity score) pooled from 5 MD runs performed for 20 or 50ns. Although no significant differences found via a student unpaired, two tailed t-test, CTII was observed to bind more avidly and faster than CTI for each of the four independent metrics analyzed.
